# The type-reproduction number of sexually transmitted infections through heterosexual and vertical transmission

**DOI:** 10.1038/s41598-019-53841-8

**Published:** 2019-11-22

**Authors:** Hiromu Ito, Taro Yamamoto, Satoru Morita

**Affiliations:** 1 Department of International Health and Medical Anthropology, Institute of Tropical Medicine, Nagasaki University, Nagasaki, 852-8523 Japan; 2 Department of Environmental Sciences, Zoology, University of Basel, 4051 Basel, Switzerland; 3 Department of Mathematical and Systems Engineering, Shizuoka University, Hamamatsu, Shizuoka 432-8561 Japan; 4 Department of Environment and Energy Systems, Graduate School of Science and Technology, Shizuoka University, Hamamatsu, Shizuoka 432-8561 Japan

**Keywords:** Statistical physics, thermodynamics and nonlinear dynamics, Complex networks, Infectious diseases

## Abstract

Multiple sexually transmitted infections (STIs) have threatened human health for centuries. Most STIs spread not only through sexual (horizontal) transmission but also through mother-to-child (vertical) transmission. In a previous work (Ito *et al*. 2019), we studied a simple model including heterosexual and mother-to-child transmission and proposed a formulation of the basic reproduction number over generations. In the present study, we improved the model to take into account some factors neglected in the previous work: adult mortality from infection, infant mortality caused by mother-to-child transmission, infertility or stillbirth caused by infection, and recovery with treatment. We showed that the addition of these factors has no essential effect on the theoretical formulation. To study the characteristics of the epidemic threshold, we derived analytical formulas for three type-reproduction numbers for adult men, adult women and juveniles. Our result indicates that if an efficient vaccine exists for a prevalent STI, vaccination of females is more effective for containment of the STI than vaccination of males, because the type-reproduction number for adult men is larger than that for adult women when they are larger than one.

## Introduction

Although sex is a private and pleasurable activity, it may result in disease^1^. Largely because of human instinct, sexually transmitted infections (STIs) have continued to exist alongside human beings for a long time. In fact, STIs were described in ancient records such as the Ebers Papyrus and the Old Testament of the Bible in Leviticus 15: 2–33^2^. STIs remain a worldwide health concern, with a global estimate of 340 million new cases of “curable” infections (e.g., syphilis, gonorrhea, chlamydia, and trichomoniasis) and millions of “incurable” infections (e.g., human immunodeficiency virus [HIV], herpes simplex viruses [HSV], human papillomaviruses [HPV], hepatitis B virus [HBV], and human T-lymphotropic virus type 1 [HTLV-1]) occurring annually among men and women aged 15–49 years^3,4^. The “incurable” viral STIs cannot be eradicated through medications that are currently available. Presently, some STIs that were formerly considered “curable” are spreading as STIs that are “incurable” because of resistance to antibiotics^5,6^.

It is difficult to accurately estimate mortality due to STIs because death certificates generally only record prevalent conditions, and STIs are rarely recorded on death certificates^7^. However, STIs contribute both directly and indirectly to human death. HIV has been well known as the virus that causes AIDS, which has a high mortality rate. In 2017, although the number of new infected people with HIV has begun to decline gradually, 36.9 million people were living with HIV/AIDS, and 940,000 people died of HIV-related illnesses, worldwide^8,9^. Hepatitis B and hepatitis C are also dangerous STIs, which increase the mortality rates from cirrhosis and liver cancer^10^. From 1990 to 2013, the estimated number of deaths caused by hepatitis, globally, increased from around 0.9 million to around 1.5 million^10^. HPV causes virtually all cervical cancer and associated cancers (i.e., oropharyngeal, vulvar, vaginal, penile, and anal cancers)^11^. Cervical cancer is among the most common cancers causing death for women in developing countries^12^. In 2018, there were 570,000 new cases of cervical cancer, worldwide, and 311,000 women died from cervical cancer^13^. Reviewing the current situation, Chesson *et al*. estimated the direct costs of major STIs at US$16.7 billion, including costs for gonorrhea, chlamydia, syphilis, trichomoniasis, hepatitis B, diseases associated with sexually transmitted HPV, genital HSV-2 infection, and HIV infection^14,15^.

In light of these concerns, mathematical models can provide ideas and knowledge that are helpful for understanding the epidemiology and control of STIs^16^. Many mathematical models have been built to understand the infection dynamics of STIs, including HIV/AIDS, syphilis, and gonorrhea^16,17^. The first mathematical model used for the explicit study of an STI (gonorrhea) was developed by Cooke and Yorke in 1973^18^. The mathematical model of HIV transmission has been extensively studied since the late 1980s^19–21^. Simple models have the advantage of analytical tractability and can be used to explain the relative merits of various prevention options. However, the real world is replete with complexities^22^.

STIs occur through sexual contact networks, which are extremely complex. The overall picture of sexual networks is unclear because information about who has sex with whom is considered extremely sensitive. We do know that human sexual networks are highly heterogeneous: Most individuals have only a few sexual partners, but a few have hundreds^23–26^. Therefore, the heterogeneity of sexual contact is an important factor of the spread of STIs.

Most STIs can also be spread vertically over generations through mother-to-child transmission. There are various types of STIs, and their transmission processes are diverse. For example, the primary route of mother-to-child (vertical) transmission of HTLV-1 is breastfeeding; intrauterine transmission and transmission via saliva are also possible infection routes, but their transmission rates are low^27^. For HTLV-1 and HBV, children who are infected through mother-to-child transmission spread STIs through sexual contact after they become adults because HTLV-1 and HBV have long latency periods before the onset of symptoms and extremely low or zero mortality among infants infected through mother-to-child transmission^28,29^. In contrast, many other STIs (e.g., congenital syphilis, neonatal herpes, congenital HIV, and many other bacterial STIs) have high infant mortality rates or other serious consequences for infants^30–34^. Clearly, these high-mortality STIs will lead to different infection dynamics, compared with low-mortality STIs.

Understanding the long-term dynamics of various STIs requires a comprehensive model that includes both horizontal and vertical transmission. In a previous study, we tested a susceptible–infected model with horizontal and vertical transmission^35^. However, the previous model assumed that the pathogenic death rate coincided with the non-pathogenic death rate, that an individual could have sexual contact soon after birth, and that infections were incurable. In the present study, we improved the previous model and constructed a comprehensive model that takes into account adult mortality from infection, infant mortality caused by mother-to-child transmission, infertility or stillbirth caused by infection, and recovery with treatment. Thus, the comprehensive model can be considered a susceptible–infected–susceptible (SIS) model. This model includes three types of individuals: juveniles, adult women, and adult men. Here, juveniles are individuals who are not mature enough to have sexual intercourse. In addition, to reproduce heterogeneous sexual contacts, we assume that each individual has their own sexual activity. In the previous study, we considered the dynamics of sexual networks^35^; however, for simplicity, we assume sexual contacts to be well mixed in the present study.

When considering homogeneous populations, epidemiologists frequently use the basic reproduction number (*R*_0_), which is the number of secondary cases that one case would produce in a completely susceptible population. However, because our model includes three types of individuals, it was convenient to calculate a type-reproduction number for each type category rather than for the population as a whole^36,37^. If the type-reproduction numbers are larger than one, the basic reproduction number is also larger than one. The type-reproduction numbers have a significant meaning from a long-term public health perspective: STIs can spread when the type-reproduction numbers are larger than one, and STIs cannot survive in the long term when the type-reproduction numbers are less than one. In this article, we derive analytical formulas for the three type-reproduction numbers using the comprehensive model we propose. We show that the type-reproduction number for adult women is qualitatively the same as the basic reproduction number proposed in our previous work^35^.

## Model

We consider a compartment model with six compartments: susceptible juveniles *S*_j_(*t*), susceptible adult female *S*_f_(*t*), susceptible adult male *S*_m_(*t*), infected juveniles *I*_j_(*t*), infected adult female *I*_f_(*t*), and infected adult male *I*_m_(*t*). These variables represent the numbers of individuals belonging to the compartments at time *t*. Juveniles cannot have sexual intercourse, whereas adults can. Moreover, we assume that each adult has different sexual activity, as a congenital attribute. The model variables and parameters are summarized in Table 1.

**Table 1:** Explanation of notation (all variables and parameters are non-negative).

Symbol	Definition
***Variables***	
*S*_j_	Number of susceptible juveniles
*S*_f_(*a*), *S*_m_(*a*)	Number of susceptible adult women and men with sexual activity *a*
*S*_f_, *S*_m_	Number of susceptible adult women and men: Eq. (7)
*I*_j_	Number of infected juveniles
*I*_f_(*a*), *I*_m_(*a*)	Number of infected adult women and men with sexual activity *a*
*I*_f_, *I*_m_	Number of infected adult women and men: Eq. (8)
*N*_f_, *N*_m_	Number of adult women and men: *S*_f_ + *I*_f_, *S*_m_ + *I*_m_
*Θ*_f_, *Θ*_m_	Probability that a sexual partner of a man or woman is infected
*k*_f_, *k*_m_	Mean number of sexual contacts per unit of time: Eq. (5)
*c*_f_, *c*_m_	Effective number of sexual contacts per unit of time: Eq. (6)
***Parameters***	
*a*	Sexual activity of each individual
*p*_f_(*a*), *p*_m_(*a*)	Distribution of sexual activity of women and men
*a*	Mean sexual activity: *a* = 1
*B*	Number of births per unit of time
*α*	vertical transmission rate from mother to infant (0 ≤ *α* ≤ 1)
*α*_eff_	vertical transmission from mother to infant (0 ≤ *α*_eff_ ≤ 1): Eq. (15)
*δ*	Rate of infertility or stillbirth caused by infection (0 ≤ *δ* ≤ 1)
*λ*, *λ*'	Maturing rate for susceptible juveniles and infected juveniles
*μ*_j_, ${\mu }_{{\rm{j}}}^{\text{'}}$	Natural and pathogenic mortality rates of juveniles
*μ*_f_, ${\mu }_{{\rm{f}}}^{\text{'}}$	Natural and pathogenic mortality rates of adult women
*μ*_m_, ${\mu }_{{\rm{m}}}^{\text{'}}$	Natural and pathogenic mortality rates of adult men
*η*_j_, *η*_f_, *η*_m_	Recovery rates of juveniles, adult women, and adult men
*γ*	Proportion of men at the time of coming of age (0 < *γ* < 1)
*β*_m→f_	Sexual transmission rate from male to female (0 < *β*_m→f_ < 1)
*β*_f→m_	Sexual transmission rate from female to male (0 < *β*_f→m_ < 1)

### Dynamics of juveniles

The dynamics of *S*_j_(*t*) and *I*_j_(*t*) are assumed to be

1 $$\begin{array}{ccc}\frac{d{S}_{{\rm{j}}}(t)}{dt} & = & B\frac{{S}_{{\rm{f}}}(t)+(1-\alpha )(1-\delta ){I}_{{\rm{f}}}(t)}{{I}_{{\rm{f}}}(t)+{S}_{{\rm{f}}}(t)}-(\lambda +{\mu }_{{\rm{j}}}){S}_{{\rm{j}}}({\rm{t}})+{\eta }_{{\rm{j}}}{I}_{{\rm{j}}}(t),\\ \frac{d{I}_{{\rm{j}}}(t)}{dt} & = & B\frac{\alpha (1-\delta ){I}_{{\rm{f}}}(t)}{{I}_{{\rm{f}}}(t)+{S}_{{\rm{f}}}(t)}-(\lambda ^{\prime} +{\eta }_{{\rm{j}}}+{\mu }_{{\rm{j}}}^{\text{'}}){I}_{{\rm{j}}}(t).\end{array}$$

Here, *B* is the number of births per unit of time, *δ* is the rate of infertility or stillbirth, and *α* is the rate of vertical transmission from mother to infant. The parameters *λ* and *λ*′ are the maturing rates for susceptible and infected juveniles, respectively. Juveniles become adults at the maturing rate. Because the pathogenicity of infection may reduce the growth of infected juveniles, we set *λ*′ ≤ *λ*. The parameter *η*_j_ stands for the juvenile cure rate, where an infected juvenile becomes susceptible with the cure rate of *η*_j_. The pathogenic premature mortality rate ${\mu }_{{\rm{j}}}^{\text{'}}$ is at least as large as the natural premature mortality rate *μ*_j_ (thus, ${\mu }_{{\rm{j}}}^{\text{'}}\ge {\mu }_{{\rm{j}}}$). Here, the juvenile’s sex is not considered because there is no need to distinguish between sexes in the juvenile stage in this model.

### Sexual activity

We assume that each adult individual has sexual activity *a* as part of their constitution. Anderson and May defined the degree of sexual activity as the number of sexual partners per unit of time^17,21^. In contrast, in network science, the degree of sexual activity is often defined as the number sexual partners that an individual has at the same time^38^. Here, we generalize degree of sexual activity to be a real number and define it as follows^39,40^. We assume that the sexual activity levels of women and men follow the distributions *p*_f_(*a*) and *p*_m_(*a*), respectively. We set the mean sexual activity levels *a* of women and men to one without losing generality:

2 $$\langle a\rangle ={\int }_{0}^{\infty }\,a{p}_{{\rm{f}}}(a)da={\int }_{0}^{\infty }\,a{p}_{{\rm{m}}}(a)da=1.$$

The total number of sexual contacts per unit of time is assumed to be *f*(*N*_f_(*t*), *N*_m_(*t*)), where *N*_f_(*t*) = *S*_f_(*t*) + *I*_f_(*t*) and *N*_m_(*t*) = *S*_m_(*t*) + *I*_m_(*t*). Thus, the rates of having a sexual contact for a woman and a man are

3 $$af({N}_{{\rm{f}}}(t),{N}_{{\rm{m}}}(t))/{N}_{{\rm{f}}}(t),\,af({N}_{{\rm{f}}}(t),{N}_{{\rm{m}}}(t))/{N}_{{\rm{m}}}(t),$$

respectively. For example, if sexual contacts are modeled by mass action, *f*(*N*_f_(*t*), *N*_m_(*t*)) ∝ *N*_f_(*t*)*N*_m_(*t*). In this case, the rates of having a sexual contact for a woman and a man are proportional to *aN*_m_(*t*) and *aN*_f_(t), respectively. This is not very realistic because the number of sexual contacts a person has increases along with the population. Alternatively, if women dominate sexual contact, *f*(*N*_f_(*t*), *N*_m_(*t*)) ∝ *N*_f_(*t*). In this case, the rates of having a sexual contact for a woman and a man are proportional to *a* and *aN*_f_(t)/*N*_m_(*t*), respectively. This may be more suitable because men have less chance of contacting women when the number of women per men decreases. In any case, the sexual contacts are assumed to be well mixed. The average numbers of sexual contacts per unit of time for women and men are given as

4 $${k}_{{\rm{f}}}=\frac{f({N}_{{\rm{f}}}(t),{N}_{{\rm{m}}}(t))}{{N}_{{\rm{f}}}(t)},\,{k}_{{\rm{m}}}=\frac{f({N}_{{\rm{f}}}(t),{N}_{{\rm{m}}}(t))}{{N}_{{\rm{m}}}(t)},$$

respectively, because we set 〈*a*〉 = 1. Moreover, it is convenient to define the averages weighted by sexual activity:

5 $$\begin{array}{ccc}{c}_{{\rm{f}}} & = & \frac{f({N}_{{\rm{f}}}(t),{N}_{{\rm{m}}}(t))}{{N}_{{\rm{f}}}(t)}{\int }_{0}^{\infty }\,{a}^{2}{p}_{{\rm{f}}}(a)da,\\ {c}_{{\rm{m}}} & = & \frac{f({N}_{{\rm{f}}}(t),{N}_{{\rm{m}}}(t))}{{N}_{{\rm{m}}}(t)}{\int }_{0}^{\infty }\,{a}^{2}{p}_{{\rm{m}}}(a)da.\end{array}$$

These values correspond to the effective average over the distribution by degree of sexual activity, as defined by May and Anderson^21^, where *c*_f_ and *c*_m_ are not simply the mean but the mean plus the ratio of variance to the mean. High heterogeneity of sexual contacts means *c*_f_ ≫ *k*_f_ and *c*_m_ ≫ *k*_m_.

### Dynamics of adults

The number of susceptible adult women whose sexual activity is in the infinitesimal interval [*a*, *a* + *da*] is denoted as *S*_f_(*t*, *a*)*da*. The same applies to *S*_m_(*t*, *a*), *I*_f_(*t*, *a*) and *I*_m_(*t*, *a*). Thus, we have

6 $$\begin{array}{ccc}{S}_{{\rm{f}}}(t) & = & {\int }_{0}^{\infty }\,{S}_{{\rm{f}}}(t,a)da,\,{S}_{{\rm{m}}}(t)={\int }_{0}^{\infty }\,{S}_{{\rm{m}}}(t,a)da,\,\\ {I}_{{\rm{f}}}(t) & = & {\int }_{0}^{\infty }\,{I}_{{\rm{f}}}(t,a)da,\,{I}_{{\rm{m}}}(t)={\int }_{0}^{\infty }\,{I}_{{\rm{m}}}(t,a)da.\end{array}$$

The dynamics of *S*_f_(*t*, *a*), *I*_f_(*t*, *a*), *S*_m_(*t*, *a*) and *I*_m_(*t*, *a*) are expressed as

7 $$\begin{array}{ccc}\frac{{\rm{\partial }}{S}_{{\rm{f}}}(t,a)}{{\rm{\partial }}t} & = & \lambda (1-\gamma ){S}_{{\rm{j}}}(t){p}_{{\rm{f}}}(a)-{\mu }_{{\rm{f}}}{S}_{{\rm{f}}}(t,a)+{\eta }_{{\rm{f}}}{I}_{{\rm{f}}}(t,a)\\  &  & -\,a{\beta }_{{\rm{m}}\to {\rm{f}}}{S}_{{\rm{f}}}(t,a){{\rm{\Theta }}}_{{\rm{m}}}(t)\frac{f({N}_{{\rm{f}}}(t),{N}_{{\rm{m}}}(t))}{{N}_{{\rm{f}}}(t)},\\ \frac{{\rm{\partial }}{S}_{{\rm{m}}}(t,a)}{{\rm{\partial }}t} & = & \lambda \gamma {S}_{{\rm{j}}}(t){p}_{{\rm{m}}}(a)-{\mu }_{{\rm{m}}}{S}_{{\rm{m}}}(t,a)+{\eta }_{{\rm{m}}}{I}_{{\rm{m}}}(t,a)\\  &  & -\,a{\beta }_{{\rm{f}}\to {\rm{m}}}{S}_{{\rm{m}}}(t,a){{\rm{\Theta }}}_{{\rm{f}}}(t)\frac{f({N}_{{\rm{f}}}(t),{N}_{{\rm{m}}}(t))}{{N}_{{\rm{m}}}(t)},\\ \frac{{\rm{\partial }}{I}_{{\rm{f}}}(t,a)}{{\rm{\partial }}t} & = & {\lambda }^{^{\prime} }(1-\gamma ){I}_{{\rm{j}}}(t){p}_{{\rm{f}}}(a)-({\mu }_{{\rm{f}}}^{^{\prime} }+{\eta }_{{\rm{f}}}){I}_{{\rm{f}}}(t,a)\\  &  & +\,a{\beta }_{{\rm{m}}\to {\rm{f}}}{S}_{{\rm{f}}}(t,a){{\rm{\Theta }}}_{{\rm{m}}}(t)\frac{f({N}_{{\rm{f}}}(t),{N}_{{\rm{m}}}(t))}{{N}_{{\rm{f}}}(t)},\\ \frac{{\rm{\partial }}{I}_{{\rm{m}}}(t,a)}{{\rm{\partial }}t} & = & {\lambda }^{^{\prime} }\gamma {I}_{{\rm{j}}}(t){p}_{{\rm{m}}}(a)-({\mu }_{{\rm{m}}}^{^{\prime} }+{\eta }_{{\rm{m}}}){I}_{{\rm{m}}}(t,a)\\  &  & +\,a{\beta }_{{\rm{f}}\to {\rm{m}}}{S}_{{\rm{m}}}(t,a){{\rm{\Theta }}}_{{\rm{f}}}(t)\frac{f({N}_{{\rm{f}}}(t),{N}_{{\rm{m}}}(t))}{{N}_{{\rm{m}}}(t)}.\end{array}$$

The parameter *γ* in the first terms on the right hand side of the above equations represents the proportion of men at the time of coming of age. If *γ* = 0.5, the same number of male and female juveniles grow to adulthood. The parameters *μ*_f_, ${\mu }_{{\rm{f}}}^{\text{'}}$, *μ*_m_, and ${\mu }_{{\rm{m}}}^{\text{'}}$ are the death rates for susceptible adult women, infected adult women, susceptible adult men, and infected adult men, respectively. Here, we set ${\mu }_{{\rm{f}}}^{\text{'}}$ ≥ *μ*_f_ and ${\mu }_{{\rm{m}}}^{\text{'}}$ ≥ *μ*_m_ because of pathogenicity. The parameters *η*_f_ and *η*_m_ are the cure rates for women and men, respectively. Note that the death and cure rates are assumed not to be directly dependent on sexual activity *a*. If *η*_j_ = *η*_f_ = *η*_m_ = 0, the infection is incurable, meaning that the model is of the susceptible–infected type. The probabilities of transmission per sexual contact are *β*_m→f_ and *β*_f→m_ for male-to-female and female-to-male transmission, respectively (0 ≤ *β*_m→f_, *β*_f→m_ ≤ 1). Here, the variables *Θ*_f_(*t*) and *Θ*_m_(*t*) stand for the probabilities that the sexual partners of a man and a woman are infected, respectively. Because a sexual partner with sexual activity *a* is selected with the probability *aN*_f_(*t*, *a*)/*N*_f_(*t*) or *aN*_m_(*t*, *a*)/*N*_m_(*t*) and the probability that the sexual partner is infected is *I*_f_(*t*, *a*)/*N*_f_(*t*, *a*) or *I*_m_(*t*, *a*)/*N*_m_(*t*, *a*), we have

8 $$\begin{array}{ccc}{\Theta }_{{\rm{f}}}(t) & = & {\int }_{0}^{\infty }\,\frac{a{I}_{{\rm{f}}}(t,a)}{{N}_{{\rm{f}}}(t)}da,\\ {\Theta }_{{\rm{m}}}(t) & = & {\int }_{0}^{\infty }\,\frac{a{I}_{{\rm{m}}}(t,a)}{{N}_{{\rm{m}}}(t)}da.\end{array}$$

## Results

### Disease-free case

First, we consider the disease-free case (*I*_j_ = *I*_f_ = *I*_m_ = *Θ*_f_ = *Θ*_m_ = 0). The dynamics of population are rewritten as

9 $$\begin{array}{ccc}\frac{d{S}_{{\rm{j}}}(t)}{dt} & = & B-(\lambda +{\mu }_{{\rm{j}}}){S}_{{\rm{j}}}({\rm{t}}),\\ \frac{{\rm{\partial }}{S}_{{\rm{f}}}(t,a)}{{\rm{\partial }}t} & = & \lambda (1-\gamma ){S}_{{\rm{j}}}(t){p}_{{\rm{f}}}(a)-{\mu }_{{\rm{f}}}{S}_{{\rm{f}}}(t,a),\\ \frac{{\rm{\partial }}{S}_{{\rm{m}}}(t,a)}{{\rm{\partial }}t} & = & \lambda \gamma {S}_{{\rm{j}}}(t){p}_{{\rm{m}}}(a)-{\mu }_{{\rm{m}}}{S}_{{\rm{m}}}(t,a).\end{array}$$

There is a stable equilibrium state

10 $${\tilde{S}}_{{\rm{j}}}=\frac{B}{\lambda +{\mu }_{{\rm{j}}}},\,{\tilde{S}}_{{\rm{f}}}(a)=\frac{\lambda B(1-\gamma )}{{\mu }_{{\rm{f}}}(\lambda +{\mu }_{{\rm{j}}})}{p}_{{\rm{f}}}(a),\,{\tilde{S}}_{{\rm{m}}}(a)=\frac{\lambda B\gamma }{{\mu }_{{\rm{m}}}(\lambda +{\mu }_{{\rm{j}}})}{p}_{{\rm{m}}}(a),$$

where the tildes over the variables indicate that the values are for the disease-free equilibrium. In this case, from Eq. (4), we have

11 $$\frac{{k}_{{\rm{f}}}}{{k}_{{\rm{m}}}}=\frac{{\tilde{N}}_{{\rm{m}}}}{{\tilde{N}}_{{\rm{f}}}}=\frac{{\tilde{S}}_{{\rm{m}}}}{{\tilde{S}}_{{\rm{f}}}}=\frac{{\mu }_{{\rm{f}}}\gamma }{{\mu }_{{\rm{m}}}(1-\gamma )}.$$

### Linearization

To derive the basic reproduction number, we linearize Eqs. (1) and (7) near the disease-free case—that is, we consider only the first order of *I*_j_, *I*_f_(*a*), *I*_m_(*a*), *Θ*_f_, *Θ*_m_ and replace ${S}_{{\rm{j}}}={\tilde{S}}_{{\rm{j}}},\,{S}_{{\rm{f}}}(a)={\tilde{S}}_{{\rm{f}}}(a),\,{S}_{{\rm{m}}}(a)={\tilde{S}}_{{\rm{m}}}(a)$ in Eqs. (1) and (7).

12 $$\begin{array}{ccc}\frac{d{I}_{{\rm{j}}}(t)}{dt} & = & \frac{\alpha (1-\delta ){\mu }_{{\rm{f}}}(\lambda +{\mu }_{{\rm{j}}})}{\lambda (1-\gamma )}{I}_{{\rm{f}}}(t)-({\lambda }^{{\rm{^{\prime} }}}+{\eta }_{{\rm{j}}}+{\mu }_{j}^{^{\prime} }){I}_{{\rm{j}}}(t),\\ \frac{{\rm{\partial }}{I}_{{\rm{f}}}(t)}{{\rm{\partial }}t} & = & {\lambda }^{{\rm{^{\prime} }}}(1-\gamma ){I}_{{\rm{j}}}(t)-({\mu }_{{\rm{f}}}^{^{\prime} }+{\eta }_{{\rm{f}}}){I}_{{\rm{f}}}(t)+{\beta }_{{\rm{m}}\to {\rm{f}}}\frac{\lambda B(1-\gamma )}{{\mu }_{{\rm{f}}}(\lambda +{\mu }_{{\rm{j}}})}{k}_{{\rm{f}}}{\Theta }_{{\rm{m}}}(t),\\ \frac{{\rm{\partial }}{I}_{{\rm{m}}}(t)}{{\rm{\partial }}t} & = & {\lambda }^{{\rm{^{\prime} }}}\gamma {I}_{{\rm{j}}}(t)-({\mu }_{{\rm{m}}}^{^{\prime} }+{\eta }_{{\rm{m}}}){I}_{{\rm{m}}}(t)+{\beta }_{{\rm{f}}\to {\rm{m}}}\frac{\lambda B\gamma }{{\mu }_{{\rm{m}}}(\lambda +{\mu }_{{\rm{j}}})}{k}_{{\rm{m}}}{\Theta }_{{\rm{f}}}(t),\\ \frac{{\rm{\partial }}{\theta }_{{\rm{f}}}(t)}{{\rm{\partial }}t} & = & {\lambda }^{{\rm{^{\prime} }}}\frac{{\mu }_{{\rm{f}}}(\lambda +{\mu }_{{\rm{j}}})}{\lambda B}{I}_{{\rm{j}}}(t)-({\mu }_{{\rm{f}}}^{^{\prime} }+{\eta }_{{\rm{f}}}){\theta }_{{\rm{f}}}(t)+{\beta }_{{\rm{m}}\to {\rm{f}}}{c}_{{\rm{f}}}{\Theta }_{{\rm{m}}}(t),\\ \frac{{\rm{\partial }}{\theta }_{{\rm{m}}}(t)}{{\rm{\partial }}t} & = & {\lambda }^{{\rm{^{\prime} }}}\frac{{\mu }_{{\rm{m}}}(\lambda +{\mu }_{{\rm{j}}})}{\lambda B}{I}_{{\rm{j}}}(t)-({\mu }_{{\rm{m}}}^{^{\prime} }+{\eta }_{{\rm{m}}}){\theta }_{{\rm{m}}}(t)+{\beta }_{{\rm{f}}\to {\rm{m}}}{c}_{{\rm{m}}}{\Theta }_{{\rm{f}}}(t).\end{array}$$

### Case without sexual transmission

Here, we consider a simple case where there is only mother-to-child transmission (*β*_m→f_ = *β*_f→m_ = 0). In this case, Eq. (12) is simplified to a three-dimensional system:

13 $$\begin{array}{ccc}\frac{d{I}_{{\rm{j}}}(t)}{dt} & = & \frac{\alpha (1-\delta ){\mu }_{{\rm{f}}}(\lambda +{\mu }_{{\rm{j}}})}{\lambda (1-\gamma )}{I}_{{\rm{f}}}(t)-({\lambda }^{^{\prime} }+{\eta }_{{\rm{j}}}+{\mu }_{{\rm{j}}}^{^{\prime} }){I}_{{\rm{j}}}(t),\\ \frac{{\rm{\partial }}{I}_{{\rm{f}}}(t)}{{\rm{\partial }}t} & = & {\lambda }^{^{\prime} }(1-\gamma ){I}_{{\rm{j}}}(t)-({\mu }_{{\rm{f}}}^{^{\prime} }+{\eta }_{{\rm{f}}}){I}_{{\rm{f}}}(t),\\ \frac{{\rm{\partial }}{I}_{{\rm{m}}}(t)}{{\rm{\partial }}t} & = & {\lambda }^{^{\prime} }\gamma {I}_{{\rm{j}}}(t)-({\mu }_{{\rm{m}}}^{^{\prime} }+{\eta }_{{\rm{m}}}){I}_{{\rm{m}}}(t).\end{array}$$

In this case, we do not need to consider the dynamics of *θ*_f_(*t*) and *θ*_m_(*t*) because they do not affect *I*_j_(*t*), *I*_f_(*t*) or *I*_m_(*t*). We write the linearized system Eq. (13) in the form $\dot{x}=(T+Q)x$, where matrix *T* corresponds to transmissions and matrix *Q* to transitions:

14 $$\begin{array}{rcl}T & = & (\begin{array}{ccc}0 & \frac{\alpha (1-\delta ){\mu }_{{\rm{f}}}(\lambda +{\mu }_{{\rm{j}}})}{\lambda (1-\gamma )} & 0\\ \frac{\alpha (1-\delta ){\mu }_{{\rm{f}}}(\lambda +{\mu }_{{\rm{j}}})}{\lambda (1-\gamma )} & 0 & 0\\ \frac{\alpha (1-\delta ){\mu }_{{\rm{f}}}(\lambda +{\mu }_{{\rm{j}}})}{\lambda (1-\gamma )} & 0 & 0\end{array}),\\ Q & = & (\begin{array}{ccc}-({\lambda }^{\text{'}}+{\eta }_{{\rm{j}}}+{\mu }_{j}^{\text{'}}) & 0 & 0\\ 0 & -\frac{\alpha (1-\delta ){\mu }_{{\rm{f}}}(\lambda +{\mu }_{{\rm{j}}})}{\lambda (1-\gamma )} & 0\\ 0 & 0 & -({\mu }_{{\rm{f}}}^{\text{'}}+{\eta }_{{\rm{f}}})\end{array}).\end{array}$$

Then, the spectral radius (dominant eigenvalue) of the next generation matrix −*TQ*^−1^ gives the reproduction number^41,42^. After some elementary algebra, we obtain the basic reproduction number for the case without sexual transmission.

15 $${\alpha }_{{\rm{eff}}}=\frac{\alpha (1-\delta ){\mu }_{{\rm{f}}}(\lambda +{\mu }_{{\rm{j}}}){\lambda }^{\text{'}}}{\lambda ({\mu }_{{\rm{f}}}^{\text{'}}+{\eta }_{{\rm{f}}})(\lambda ^{\prime} +{\eta }_{{\rm{j}}}+{\mu }_{{\rm{j}}}^{\text{'}})}.$$

Here, the amount *α*_eff_ is the efficient vertical transmission rate.

**Figure 1:**
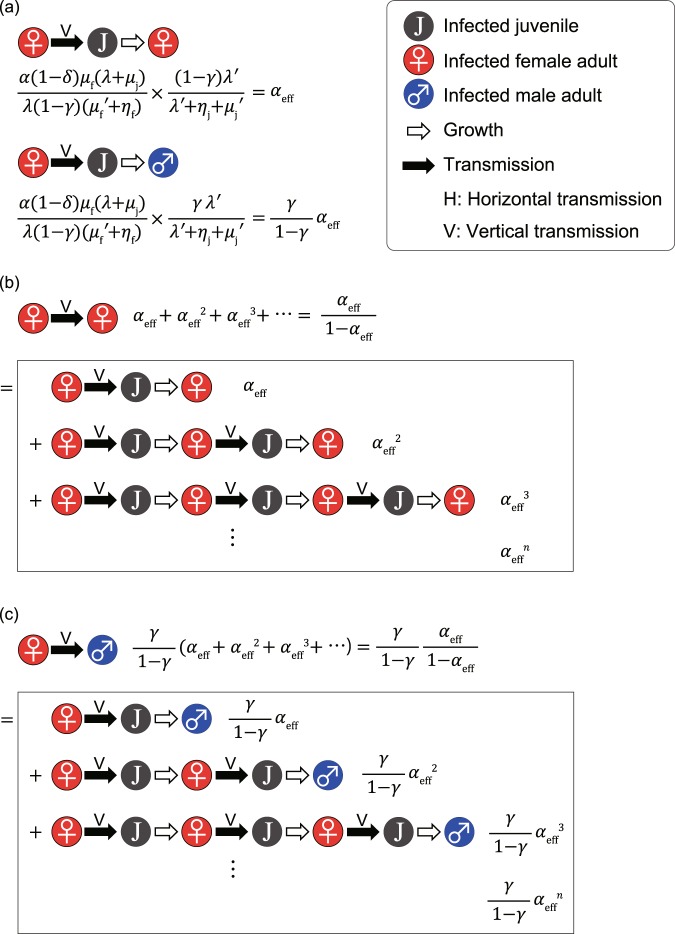
Intuitive derivation of Eqs. (15), (20–22). (**a**) The two figures represent the processes through which daughters and sons are vertically infected by an infected adult woman, where the average production numbers of vertically infected juveniles are given by *α*_eff_ and $\frac{\gamma }{1-\gamma }{\alpha }_{{\rm{eff}}}$, respectively. (**b**) The average total number of female offspring infected vertically by a horizontally infected adult woman is derived by a geometric series with initial term *α*_eff_ and geometric ratio *α*_eff_, which gives Eq. (21). (**c**) The average total number of male offspring infected vertically by a horizontally infected adult woman is derived similarly, giving Eq. (22).

Equation (15) can be intuitively understood as follows (see Fig. 1a). The number of births per unit of time in the disease-free equilibrium is

16 $$B=\frac{{\mu }_{{\rm{f}}}(\lambda +{\mu }_{{\rm{j}}})}{\lambda (1-\gamma )}{\tilde{S}}_{{\rm{f}}},$$

as is apparent from Eq. (10). Referring to Eq. (1), the average number of children born to an infected adult women per unit of time is

17 $$\frac{\alpha (1-\delta ){\mu }_{{\rm{f}}}(\lambda +{\mu }_{{\rm{j}}})}{\lambda (1-\gamma )}.\,$$

Because the average duration for infected adult women is 1/(${\mu }_{{\rm{f}}}^{\text{'}}$ + *η*_f_), the average number of children born to an infected adult woman is

18 $$\frac{\alpha (1-\delta ){\mu }_{{\rm{f}}}(\lambda +{\mu }_{{\rm{j}}})}{\lambda (1-\gamma )({\mu }_{{\rm{f}}}^{^{\prime} }+{\eta }_{{\rm{f}}})}.\,$$

The probability that a juvenile survives and becomes an adult woman is

19 $$\frac{(1-\gamma )\lambda ^{\prime} }{\lambda ^{\prime} +{\eta }_{{\rm{j}}}+{\mu }_{{\rm{j}}}^{\text{'}}}.$$

The average number of daughters infected vertically by an infected adult woman is given by the product of Eqs. (18) and (19), which leads to Eq. (15). The value of *α*_eff_ represents the average number of infected adult daughters of an infected adult woman in a completely susceptible population. Thus, the epidemic threshold is given by *α*_eff_ = 1. Because *α* ≤ 1, *λ*′ ≤ *λ*, ${\mu }_{{\rm{j}}}^{\text{'}}\ge {\mu }_{{\rm{j}}}$, ${\mu }_{{\rm{f}}}^{\text{'}}$ ≥ *μ*_f_, *η*_f_ ≥ 0, *η*_j_ ≥ 0, and *δ* ≥ 0, *α*_eff_ is always less than one. Therefore, STIs cannot survive if they spread only by mother-to-child transmission. In the same way (see Fig. 1a), we can show that the average number of sons infected vertically by an infected adult woman is

20 $$\frac{\gamma }{1-\gamma }\,{\alpha }_{{\rm{eff}}}.$$

As is shown in Fig. 1b, the average number of adult women infected through consecutive mother-to-child transmissions from an infected adult woman is

21 $$\frac{{\alpha }_{{\rm{eff}}}}{1-{\alpha }_{{\rm{eff}}}}.$$

As is shown in Fig. 1c, the average number of adults men infected through consecutive mother-to-child transmissions from an infected adult woman is

22 $$\frac{\gamma }{1-\gamma }\,\frac{{\alpha }_{{\rm{eff}}}}{1-{\alpha }_{{\rm{eff}}}}.$$

### Type-reproduction numbers for the general case

Here, we calculate the type-reproduction numbers for three compartments. First, to derive the type-reproduction number for women infected horizontally, we focus on adult women infected through sexual transmission and regard vertical transmissions as transitions. We rewrite the linearized system Eq. (12) in the form $\dot{x}$ = (*T* + *Q*)*x*—that is,

23 $$\begin{array}{ccc}T & = & (\begin{array}{ccccc}0 & 0 & 0 & 0 & 0\\ 0 & 0 & 0 & 0 & {A}_{25}\\ 0 & 0 & 0 & 0 & 0\\ 0 & 0 & 0 & 0 & {A}_{45}\\ 0 & 0 & 0 & 0 & 0\end{array}),\\ Q & = & (\begin{array}{ccccc}-{A}_{11} & {A}_{12} & 0 & 0 & 0\\ {A}_{21} & -{A}_{22} & 0 & 0 & 0\\ {A}_{31} & 0 & -{A}_{33} & {A}_{34} & 0\\ {A}_{41} & 0 & 0 & -{A}_{44} & 0\\ {A}_{51} & 0 & 0 & {A}_{54} & -{A}_{55}\end{array}).\end{array}$$

Here, for example, *A*_11_ = *λ*′ + *η*_j_ + ${\mu }_{{\rm{j}}}^{\text{'}}$ and ${A}_{25}={\beta }_{{\rm{m}}\to {\rm{f}}}\frac{\lambda B(1-\gamma )}{{\mu }_{{\rm{f}}}(\lambda +{\mu }_{{\rm{j}}})}{k}_{{\rm{f}}}$. Calculating the dominant eigenvalue of −*TQ*^−1^, we obtain the type-reproduction number for adult women:

24 $${R}_{{\rm{f}}}=\frac{{\beta }_{{\rm{f}}\to {\rm{m}}}{c}_{{\rm{f}}}}{{\mu }_{{\rm{f}}}^{\text{'}}+{\eta }_{{\rm{f}}}}\frac{{\beta }_{{\rm{m}}\to {\rm{f}}}{c}_{{\rm{m}}}}{{\mu }_{{\rm{m}}}^{\text{'}}+{\eta }_{{\rm{m}}}}+\frac{{\alpha }_{{\rm{eff}}}}{1-{\alpha }_{{\rm{eff}}}}\frac{{\beta }_{{\rm{f}}\to {\rm{m}}}{k}_{{\rm{f}}}}{{\mu }_{{\rm{f}}}^{\text{'}}+{\eta }_{{\rm{f}}}}\frac{{\beta }_{{\rm{m}}\to {\rm{f}}}{c}_{{\rm{m}}}}{{\mu }_{{\rm{m}}}^{\text{'}}+{\eta }_{{\rm{m}}}}+\frac{\gamma }{1-\gamma }\frac{{\alpha }_{{\rm{eff}}}}{1-{\alpha }_{{\rm{eff}}}}\frac{{\beta }_{{\rm{m}}\to {\rm{f}}}{k}_{{\rm{m}}}}{{\mu }_{{\rm{m}}}^{\text{'}}+{\eta }_{{\rm{m}}}}.$$

**Figure 2:**
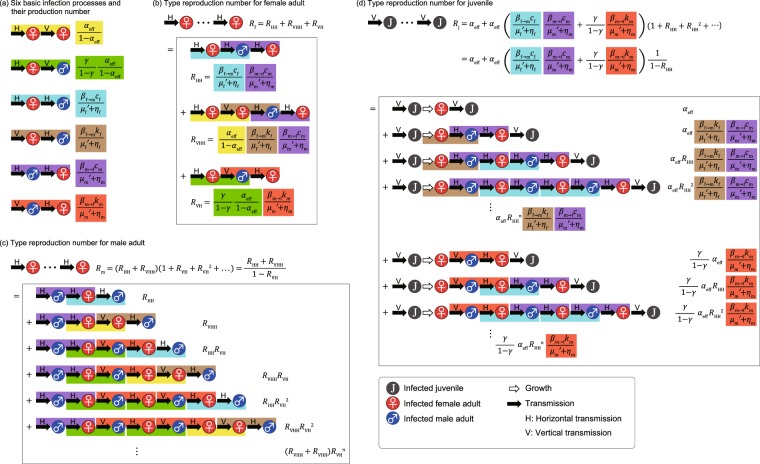
Intuitive derivation of type-reproduction numbers: Eqs. (24), (26) and (28). (**a**) There are four basic processes of infection spread starting from an infected adult woman and two starting from an infected adult man. The top two processes (yellow and green) represent vertical transmission starting from a horizontally infected adult woman, where the average production numbers are calculated as in Fig. 1b,c. Note that these processes contain vertical transmission starting from a vertically infected adult woman. The middle two processes represent horizontal transmission from a horizontally (blue) or vertically (brown) infected woman. The bottom two processes represent horizontal transmission from a horizontally (purple) or vertically (red) infected man. The average production numbers are given by the product of the transmissibility (*β*_f→m_ or *β*_m→f_), contact rate (*c*_f_, *k*_f_, *c*_m_ or *k*_m_), and duration (1/(${\mu }_{{\rm{f}}}^{\text{'}}$ + *η*_f_) or 1/(${\mu }_{{\rm{m}}}^{\text{'}}$ + *η*_m_)). (**b**) There are three paths starting from a horizontally infected adult woman and ending with a horizontally infected adult woman: two horizontal transmissions (HH), one vertical and two horizontal transmissions (VHH), and one vertical and one horizontal transmission (VH). Because the HH case is made up of the processes shown in blue and purple in (**a)**, the reproduction rate *R*_HH_ for the HH case is given by the product of their average production numbers. We can calculate the reproduction rates *R*_VHH_ and *R*_VH_ for the VHH and VH cases in the same way. Thus, the type-reproduction number for adult women is given by summing these rates: *R*_HH_ + *R*_VHH_ + *R*_VH_; thus, Eq. (24) is obtained. (**c**) The paths starting with a horizontally infected adult man and ending with a horizontally infected adult man are more complicated. The HH case (purple and blue) and the VHH case (purple, yellow, and brown) are similar to (**b**) (see the top two parts). The average production numbers of these cases are *R*_HH_ and *R*_VHH_. In addition to these two cases, secondary infected women may vertically infect their sons, who may then infect other women (i.e., we can insert the green and red parts in the way; see the middle two parts). The average production numbers of these cases are *R*_HH_*R*_VH_ and *R*_VHH_*R*_VH_. This procedure can be repeated (see the bottom two parts). Thus, the type-reproduction number for adult men is derived by a geometric series with geometric ratio *R*_VH_, and Eq. (26) is obtained. (**d**) The paths starting with an infected juvenile and ending with an infected juvenile are also complicated. The case through one woman is simple, and it is obvious that its reproduction rate is *α*_eff_. There are two cases through two women: One includes men infected horizontally (brown and purple), and the other includes those infected vertically (red). Inserting the blue and purple part in the way, we obtain two cases through three women. This procedure can be repeated. Considering two geometric series with geometric ratio *R*_HH_, we obtain the type-reproduction number for juveniles—Eq. (28).

If *R*_f_ > 1, the STI can spread over the population. Our previous study treating a simpler model gave a similar formula^35^, where we called Eq. (24) the basic reproduction number over generations. This metric is the average number of sexually infected adult women generated from a sexually infected woman in a completely susceptible population. As is shown in Fig. 2b, the first term represents the propagation through only two types of horizontal transmission (female-to-male and male-to-female); the second term represents the propagation through vertically infected women and these two types of horizontal transmission; and the third term represents the propagation through vertically infected men and male-to-female horizontal transmission. Note that the first term is dominant in Eq. (24) because *c*_m_ ≫ *k*_m_ and *c*_f_ ≫ *k*_f_ if *α*_eff_ is not close to one.

Second, focusing on adult men infected through sexual transmission, the linearized system Eq. (12) in the form $\dot{x}$ = (*T* + *Q*)*x* is given by

25 $$\begin{array}{ccc}T & = & (\begin{array}{ccccc}0 & 0 & 0 & 0 & 0\\ 0 & 0 & 0 & 0 & 0\\ 0 & 0 & 0 & {A}_{34} & 0\\ 0 & 0 & 0 & 0 & 0\\ 0 & 0 & 0 & {A}_{54} & 0\end{array}),\\ Q & = & (\begin{array}{ccccc}-\,{A}_{11} & {A}_{12} & 0 & 0 & 0\\ {A}_{21} & -\,{A}_{22} & 0 & 0 & {A}_{25}\\ {A}_{31} & 0 & -\,{A}_{33} & 0 & 0\\ {A}_{41} & 0 & 0 & -\,{A}_{44} & {A}_{45}\\ {A}_{51} & 0 & 0 & 0 & -\,{A}_{55}\end{array}).\end{array}$$

Calculating the dominant eigenvalue of −*TQ*^−1^, we obtain the type-reproduction number for adult men:

26 $${R}_{{\rm{m}}}=\frac{\frac{{\beta }_{{\rm{f}}\to {\rm{m}}}{c}_{{\rm{f}}}}{{\mu }_{{\rm{f}}}^{\text{'}}+{\eta }_{{\rm{f}}}}\frac{{\beta }_{{\rm{m}}\to {\rm{f}}}{c}_{{\rm{m}}}}{{\mu }_{{\rm{m}}}^{\text{'}}+{\eta }_{{\rm{m}}}}+\frac{{\alpha }_{{\rm{eff}}}}{1-{\alpha }_{{\rm{eff}}}}\frac{{\beta }_{{\rm{f}}\to {\rm{m}}}{k}_{{\rm{f}}}}{{\mu }_{{\rm{f}}}^{\text{'}}+{\eta }_{{\rm{f}}}}\frac{{\beta }_{{\rm{m}}\to {\rm{f}}}{c}_{{\rm{m}}}}{{\mu }_{{\rm{m}}}^{\text{'}}+{\eta }_{{\rm{m}}}}}{1-\frac{\gamma }{1-\gamma }\frac{{\alpha }_{{\rm{eff}}}}{1-{\alpha }_{{\rm{eff}}}}\frac{{\beta }_{{\rm{m}}\to {\rm{f}}}{k}_{{\rm{m}}}}{{\mu }_{{\rm{m}}}^{\text{'}}+{\eta }_{{\rm{m}}}}}.$$

We can intuitively derive Eq. (26) by enumerating all of the infection paths (see Fig. 2c). In the case of

$$\frac{\gamma }{1-\gamma }\frac{{\alpha }_{{\rm{eff}}}}{1-{\alpha }_{{\rm{eff}}}}\frac{{\beta }_{{\rm{m}}\to {\rm{f}}}{k}_{{\rm{m}}}}{{\mu }_{{\rm{m}}}^{\text{'}}+{\eta }_{{\rm{m}}}} < 1,$$

Equation (26) is meaningful, representing the average number of sexually infected adult men generated from a sexually infected man in a completely susceptible population. Otherwise, *R*_m_ diverges to infinity. It is obvious that *R*_m_ > 1 if and only if *R*_f_ > 1. Moreover, we emphasize that *R*_m_ > *R*_f_ if *R*_f_ > 1.

Finally, focusing juveniles infected through mother-to-child transmission, the linearized system Eq. (12) in the form $\dot{x}$ = (*T* + *Q*)*x* is given by

27 $$\begin{array}{ccc}T & = & (\begin{array}{ccccc}0 & {A}_{12} & 0 & 0 & 0\\ 0 & 0 & 0 & 0 & 0\\ 0 & 0 & 0 & 0 & 0\\ 0 & 0 & 0 & 0 & 0\\ 0 & 0 & 0 & 0 & 0\end{array}),\\ Q & = & (\begin{array}{ccccc}-{A}_{11} & 0 & 0 & 0 & 0\\ {A}_{21} & -{A}_{22} & 0 & 0 & {A}_{25}\\ {A}_{31} & 0 & -{A}_{33} & {A}_{34} & 0\\ {A}_{41} & 0 & 0 & -{A}_{44} & {A}_{45}\\ {A}_{51} & 0 & 0 & {A}_{54} & -{A}_{55}\end{array}).\end{array}$$

Calculating the dominant eigenvalue of −*TQ*^−1^, we obtain the type-reproduction number for adult men:

28 $${R}_{{\rm{j}}}={\alpha }_{{\rm{eff}}}(1+\frac{\frac{{\beta }_{{\rm{f}}\to {\rm{m}}}{k}_{{\rm{f}}}}{{\mu }_{{\rm{f}}}^{\text{'}}+{\eta }_{{\rm{f}}}}\frac{{\beta }_{{\rm{m}}\to {\rm{f}}}{c}_{{\rm{m}}}}{{\mu }_{{\rm{m}}}^{\text{'}}+{\eta }_{{\rm{m}}}}+\frac{\gamma }{1-\gamma }\frac{{\beta }_{{\rm{m}}\to {\rm{f}}}{k}_{{\rm{m}}}}{{\mu }_{{\rm{m}}}^{\text{'}}+{\eta }_{{\rm{m}}}}}{1-\frac{{\beta }_{{\rm{f}}\to {\rm{m}}}{c}_{{\rm{f}}}}{{\mu }_{{\rm{f}}}^{\text{'}}+{\eta }_{{\rm{f}}}}\frac{{\beta }_{{\rm{m}}\to {\rm{f}}}{c}_{{\rm{m}}}}{{\mu }_{{\rm{m}}}^{\text{'}}+{\eta }_{{\rm{m}}}}\,}).$$

We can intuitively derive Eq. (28) by enumerating all of the infection paths (see Fig. 2d). In the case of

$$\frac{{\beta }_{{\rm{f}}\to {\rm{m}}}{c}_{{\rm{f}}}}{{\mu }_{{\rm{f}}}^{\text{'}}+{\eta }_{{\rm{}}}}\frac{{\beta }_{{\rm{m}}\to {\rm{f}}}{c}_{{\rm{m}}}}{{\mu }_{{\rm{m}}}^{\text{'}}+{\eta }_{{\rm{m}}}} < 1,$$

Equation (28) is meaningful because it represents the average number of vertically infected juveniles generated from a vertically infected juvenile in a completely susceptible population. If sexual transmission is frequent enough (i.e., $\frac{{\beta }_{{\rm{f}}\to {\rm{m}}}{c}_{{\rm{f}}}}{{\mu }_{{\rm{f}}}^{\text{'}}+{\eta }_{{\rm{f}}}}\frac{{\beta }_{{\rm{m}}\to {\rm{f}}}{c}_{{\rm{m}}}}{{\mu }_{{\rm{m}}}^{\text{'}}+{\eta }_{{\rm{m}}}} > 1$), *R*_j_ diverges to infinity. The divergence of *R*_j_ means that the spread of the STI cannot be stopped even if we eliminate mother-to-child transmission completely. It is obvious that *R*_j_ > 1 if and only if *R*_f_ > 1.

The type-reproduction numbers in Eqs. (24), (26) and (28) are independent of the number of births per unit of time *B*. Thus, regardless of the details of the birth process, these formulas are always valid for the equilibrium. Without mother-to-child transmission (*α* = 0 or *α*_eff_ = 0), we obtain

29 $${R}_{{\rm{f}}}={R}_{{\rm{m}}}=\frac{{\beta }_{{\rm{f}}\to {\rm{m}}}{c}_{{\rm{f}}}}{{\mu }_{{\rm{f}}}^{\text{'}}+{\eta }_{{\rm{f}}}}\frac{{\beta }_{{\rm{m}}\to {\rm{f}}}{c}_{{\rm{m}}}}{{\mu }_{{\rm{m}}}^{\text{'}}+{\eta }_{{\rm{m}}}},$$

which agrees with May and Anderson’s result for heterosexual transmission, where they presented ${R}_{0}=\sqrt{{\beta }_{{\rm{f}}\to {\rm{m}}}{c}_{{\rm{f}}}{\beta }_{{\rm{m}}\to {\rm{f}}}{c}_{{\rm{m}}}}$ when *η*_f_ = *η*_m_ = 0 and ${\mu }_{{\rm{f}}}^{\text{'}}$ = ${\mu }_{{\rm{m}}}^{\text{'}}$ = 1^21^. In this case, *R*_f_ and *R*_m_ coincide with the square of the conventional *R*_0_.

### Graphical analysis

**Figure 3:**
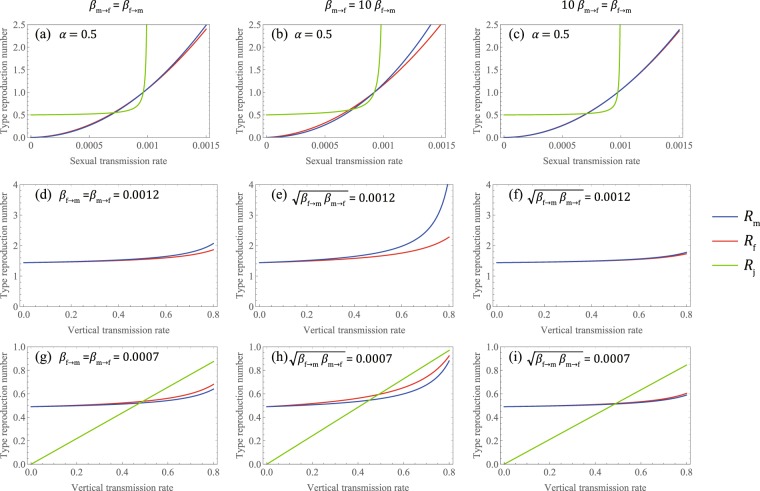
Graphical analysis of the three type-reproduction numbers. (**a**–**c**) The three type-reproduction numbers (*R*_m_, *R*_f_, *R*_j_) are plotted as a function of the geometric mean of the sexual transmission rates $\sqrt{{\beta }_{{\rm{f}}\to {\rm{m}}}{\beta }_{{\rm{m}}\to {\rm{f}}}}$ for constant vertical transmission rate (*α* = 0.5), and **(d–i)** they are plotted as a function of *α* for constant sexual transmission rates. The left panels (**a**,**d**,**g**) represent the same sexual transmission rate (*β*_f→m_ = *β*_m→f_), the middle panels (**b**,**e**,**h**) represent the case in which the sexual transmission rate from males to females is greater than that in the opposite direction (*β*_m→f_ = 10*β*_f→m_), and the right panels (**c**,**f**,**i**) represent the case in which the sexual transmission rate from females to males is greater than that in the opposite direction (*β*_f→m_ = 10*β*_m→f_). For the case that *β*_f→m_*β*_m→f_ is large **(d–f)**, *R*_j_ is not plotted because it diverges. The other parameters are set as *k*_f_, *k*_m_ = 0.8, *c*_f_, *c*_m_ = 20, *μ*_f_, ${\mu }_{{\rm{f}}}^{\text{'}}$, *μ*_m_, ${\mu }_{{\rm{m}}}^{\text{'}}$, *μ*_j_, ${\mu }_{{\rm{j}}}^{\text{'}}$ = 1/50, *η*_f_, *η*_m_, *η*_j_ = 0, *δ* = 0, *λ* = *λ*′ = 1/15, then *α*_eff_ = *α*.

In Fig. 3, we show the relationship between the infection rate (vertical and horizontal) and the three type of reproduction numbers. Here, we set *k*_f_, *k*_m_ = 0.8, *c*_f_, *c*_m_ = 20, *μ*_f_, ${\mu }_{{\rm{f}}}^{\text{'}}$, *μ*_m_, ${\mu }_{{\rm{m}}}^{\text{'}}$, *μ*_j_, ${\mu }_{{\rm{j}}}^{\text{'}}$ = 1/50, *η*_f_, *η*_m_, *η*_j_ =0, *δ* = 0, and *λ* = *λ*′ = 1/15, and then *α*_eff_ = *α*. When the vertical transmission rate is low or *β*_m→f_≪*β*_f→m_, there is almost no difference between *R*_f_ and *R*_m_. Otherwise, when STI is not widespread, the type-reproduction number of females exceeds that of males (i.e., 1 > *R*_f_ > *R*_m_); when the STI has already spread, the type-reproduction number of males is larger than that of females (i.e., *R*_m_ > *R*_f_ > 1). This result means that if an efficient vaccine for the STI exists, vaccination of females is more effective for containment of the STI than vaccination of males because herd immunity would be possible at vaccination rates above 1 − 1/*R*_f_ for females or 1 − 1/*R*_m_ for males.

As far as the type-reproduction numbers are concerned, the effective increase in the cure rate *η*_m_ is equivalent to the effective decrease in *β*_m→f_. Similarly, the increase in mortality ${\mu }_{{\rm{m}}}^{\text{'}}$ due to the STI is equivalent to the decrease in *β*_m→f_. On the other hand, the increase in cure rate *η*_f_ is equivalent to the decrease in both *β*_f→m_ and *α*_eff_, and the increase in mortality ${\mu }_{{\rm{f}}}^{\text{'}}$ is equivalent to the decrease in both *β*_f→m_ and *α*_eff_. Equations (24), (26) and (28) do not explicitly include the mortality rate of juveniles (*μ*_j_,${\mu }_{{\rm{j}}}^{\text{'}}$), but the type-reproduction numbers depend on *μ*_j_ and ${\mu }_{{\rm{j}}}^{\text{'}}$ through the efficient vertical transmission rate *α*_eff_, which decreases when ${\mu }_{{\rm{j}}}^{\text{'}}$ increases. Keeping *c*_f_/*k*_f_ (*c*_m_/*k*_m_) constant, increasing *k*_f_ (*k*_m_) corresponds to increasing *β*_f→m_ (*β*_m→f_).

## Discussion

In this article, we have presented a compartment model of STIs, considering both mother-to-child transmission and sexual transmission. The proposed model is of major importance because it takes into account the heterogeneity of sexual contacts, adult mortality from infection, infant mortality caused by mother-to-child transmission, infertility or stillbirth caused by infection, and recovery with treatment. Our model derives analytical formulas for the type-reproduction numbers *R*_f_, *R*_m_, and *R*_j_. Because these metrics give the same epidemic threshold, it is convenient to use the simplest formula, Eq. (24). Equation (24) coincides qualitatively with the basic production number over generations that we proposed in previous work^35^.

The model proposed here allows us to understand how the various effects of mother-to-child transmission on juveniles will change the epidemic threshold. It should be emphasized that all effects of STIs on the juvenile period before reproductive age are expressed mathematically in *α*_eff_. For example, infant mortality and various disturbances to growth reduce *α*_eff_. Because *α*_eff_ is not in the dominant term in Eq. (24), *α*_eff_ does not strongly affect the epidemic threshold. In other words, the spreading efficiency of STIs will not be strongly altered even if various problems in the juvenile stage (i.e., mortality or stillbirth) are solved. In sum, we suggest that the heterogeneity of sexual contacts highly contributes to the spread of STIs, and mother-to-child transmission may work as an auxiliary infection route, contributing to the survival of STIs. This fact was derived from the mathematical analysis and is consistent with our previous work, which did not consider mortality from STIs^35^.

The analytical formulas for type-reproduction numbers can provide us with important insight into strategies to prevent the spreading of STIs. If there was an efficient vaccine, herd immunity would be possible at vaccination rates above 1 − 1/*R*_f_ for women or 1 − 1/*R*_m_ for men. *R*_m_ > *R*_f_, as was shown above, because of mother-to-child transmission; therefore, our result means that if the number of vaccines and the amount of funds are limited, it is more efficient to concentrate the vaccine in women only. For example, in many countries, publicly funded HPV immunization programs target young adolescent girls, who are at the border between the juvenile period and adulthood^43^. According to our results, this intensive vaccination investment in young girls makes sense mathematically. To apply our results quantitatively to actual STIs, we need to estimate the model parameters using clinical epidemiological and demographic data on sexual behavior. However, it is difficult to estimate the infection rate (i.e., *β*_m→f_, *β*_f→m_, and *α*) and mortality rate (i.e., ${\mu }_{{\rm{j}}}^{\text{'}}$, ${\mu }_{{\rm{f}}}^{\text{'}}$, and ${\mu }_{{\rm{m}}}^{\text{'}}$) of specific individual STIs because people frequently have more than one STI at the same time^44^. In addition, the data on the distribution of human sexual activity are insufficient^45^.

Nevertheless, we are able to form some preliminary qualitative conclusions. Many STIs have serious fetal consequences, such as TORCH infections^46,47^, which include toxoplasmosis, other diseases (syphilis, varicella-zoster, parvovirus), rubella, cytomegalovirus, and herpes infections. Most TORCH infections cause mild maternal morbidity and have serious fetal consequences, and the treatment of maternal infections often has no impact on fetal outcomes; thus, the recovery rate *η*_j_ for juveniles is nearly zero^47^. For incurable STIs such as HIV, HSV, HPV, HTLV-1, and antibiotic-resistant STIs, the proposed model is a susceptible–infected model (*η*_j_ = *η*_f_ = *η*_m_ = 0). For recovery with treatment, the recovery rates depend on the medical systems and treatment strategy. For example, it is possible that infected women are less aware of their infections than are infected men with the same STIs, *η*_f_ < *η*_m_ because the treatment of infected women and is not promoted to the same extent as is the treatment of infected men^48,49^. In addition, some STIs may inhibit children’s growth even when the infections are not fatal. For example, HSV can bring on central nervous system disorder^30^, and cytomegalovirus can lead to long-term neurological sequelae including unilateral and bilateral sensorineural hearing loss, mental retardation, cerebral palsy, and impaired vision from chorioretinitis^50–52^. In these cases, the maturing rate *λ*' for infected juveniles is lower than the maturing rate *λ* for susceptible juveniles.

The model proposed here did not consider some important factors that are related to the diffusion of STIs. First, we neglected some routes of transmission, such as needle sharing^53–55^ and blood transfusion^56^, because these routes were rare in ancient times and there are many efforts to reduce them now^57,58^. Moreover, only a few STIs are known to be transmitted by mosquito bites^59^. Here, we focused on understanding the diffusion contribution of sexual and mother-to-child transmission. Second, our model assumed that sexual contacts are well mixed, and the effects of sex workers^55^, marital status and age structure were not taken into account. The presence of sex workers will increase the heterogeneity of sexual contact. Marital status will affect the infection dynamics of STIs because marriage yields sustained sexual activity with a specific partner^60^. The age structure also will influence the diffusion of STIs^61^. Sexual transmission tends to occur among people of the same generation, whereas mother-to-child transmission propagates infections across generations; thus, our model may slightly underestimate the contribution of mother-to-child transmission. Third, homosexual contact was not considered here, although homosexual transmission is important, especially for the spread not of only HIV but also of HBV, syphilis etc.^62^. In this article, our aim was to understand the complex infection dynamics, simultaneously considering both unequal sexual transmission rates between males and females and mother-to-child transmission. If we also considered homosexual and bisexual networks, the infection dynamics would become extremely complicated. Thus, here, we have not discussed the spread of STIs through homosexual networks, such as men who have sex with men. Fourth, we have assumed that sexual activity is not inherited and does not depend on whether an individual is infected or not. In addition, we did not consider the correlation between sexual activity and fecundity. Constructing a model that takes into account the inheritance of sexual activity may reproduce the heterogeneous distribution of this variable. These limitations should be addressed in future research.

In conclusion, the comprehensive model proposed in this article can clarify the complex transmission of STIs. This model is prospective: It is meant to predict the spread of various STIs. We derived analytical formulas for three type-reproduction numbers, *R*_f_, *R*_m_, and *R*_j_, and elucidated the relationships among them. However, the quantitative evaluation of these metrics for actual STIs remains a topic for future research. The quantitative application of our model has the potential to clarify which kinds of countermeasures will be effective in combating STIs.

## Data Availability

The authors declare that all data supporting the findings of this study are available within the article or from the corresponding author upon reasonable request.
